# Epstein–Barr Virus MicroRNAs Are Evolutionarily Conserved and Differentially Expressed

**DOI:** 10.1371/journal.ppat.0020023

**Published:** 2006-03-24

**Authors:** Xuezhong Cai, Alexandra Schäfer, Shihua Lu, John P Bilello, Ronald C Desrosiers, Rachel Edwards, Nancy Raab-Traub, Bryan R Cullen

**Affiliations:** 1 Center for Virology and Department of Molecular Genetics and Microbiology, Duke University Medical Center, Durham, North Carolina, United States of America; 2 New England Primate Research Center, Harvard Medical School, Southborough, Massachusetts, United States of America; 3 Department of Microbiology and Immunology and Lineberger Comprehensive Cancer Center, University of North Carolina at Chapel Hill, Chapel Hill, North Carolina, United States of America; University of California San Francisco, United States of America

## Abstract

The pathogenic lymphocryptovirus Epstein–Barr virus (EBV) is shown to express at least 17 distinct microRNAs (miRNAs) in latently infected cells. These are arranged in two clusters: 14 miRNAs are located in the introns of the viral *BART* gene while three are located adjacent to *BHRF1.* The *BART* miRNAs are expressed at high levels in latently infected epithelial cells and at lower, albeit detectable, levels in B cells. In contrast to the tissue-specific expression pattern of the *BART* miRNAs, the *BHRF1* miRNAs are found at high levels in B cells undergoing stage III latency but are essentially undetectable in B cells or epithelial cells undergoing stage I or II latency. Induction of lytic EBV replication was found to enhance the expression of many, but not all, of these viral miRNAs. Rhesus lymphocryptovirus, which is separated from EBV by ≥13 million years of evolution, expresses at least 16 distinct miRNAs, seven of which are closely related to EBV miRNAs. Thus, lymphocryptovirus miRNAs are under positive selection and are likely to play important roles in the viral life cycle. Moreover, the differential regulation of EBV miRNA expression implies distinct roles during infection of different human tissues.

## Synopsis

Vertebrate cells express a large family of diverse small RNAs, called microRNAs, that can inhibit the expression of specific target genes. Recently, it has become apparent that several pathogenic human viruses, and in particular herpes viruses, also encode microRNAs that these viruses likely use to prevent infected cells and individuals from mounting effective antiviral responses. Here, we demonstrate that Epstein–Barr virus (EBV), which causes infectious mononucleosis and also some cancers in humans, makes 17 different microRNAs in infected human cells. These microRNAs are found in two clusters in the viral genome, one of three microRNAs, the second of 14 microRNAs, that are differentially expressed in different kinds of EBV-induced human tumors. Analysis of the closely related rhesus lymphocryptovirus shows that seven of these EBV microRNAs have been conserved in this simian virus across >13 million years of divergent evolution. This argues that these microRNAs likely play an important role in EBV replication and represents the first demonstration of the evolutionary conservation of viral microRNAs.

## Introduction

MicroRNAs (miRNAs) are small noncoding RNAs, generally 21–24 nt in length, that can posttranscriptionally down-regulate the expression of mRNAs bearing complementary target sequences [1]. Over 300 miRNAs have been identified in humans, and comparable numbers are expressed in all metazoan eukaryotes analyzed thus far. Although relatively few mRNA targets for specific miRNAs have been identified in vertebrates, experiments in plants, *Caenorhabditis elegans, Drosophila,* and zebra fish indicate that miRNAs play a critical role in the appropriate regulation of gene expression during the differentiation and development of metazoan organisms [1–7].

miRNAs are closely related to small interfering RNAs, approximately 22-nt-long noncoding RNAs that are generated by cleavage of double-stranded RNAs by the RNase III enzyme Dicer [1]. In plants and in invertebrates, small interfering RNAs generated from double-stranded RNAs produced during viral replication have been shown to play an important role in the innate immune response of these organisms to viral infection by inducing an RNA interference response specific for the infecting virus [8,9]. While it was therefore initially proposed that a virus-induced RNA interference response might also be important in allowing vertebrate species to attenuate virus replication, evidence obtained so far has not supported this hypothesis [10]. However, a number of viruses have been shown to encode miRNAs that are believed to play a potentially critical role in the viral life cycle in vivo. Thus, the herpesviruses Epstein–Barr virus (EBV), Kaposi sarcoma-associated herpesvirus (KSHV), human cytomegalovirus, and mouse herpesvirus 68 have previously been reported to encode five, eleven, nine, and nine miRNAs, respectively [10–13]. Moreover, the unrelated DNA tumorvirus SV40 encodes at least one miRNA [14]. In the case of the EBV miRNA miR-BART2 and the SV40 miRNA, it has been proposed that these viral miRNAs down-regulate the expression of a virus-encoded mRNA [11,14]. In contrast, mRNA targets for the other viral miRNAs have yet to be identified, although several host mRNAs have been proposed [11,12]. It has been hypothesized that these herpesvirus miRNAs, which are all expressed in latently infected cells, may facilitate the viral life cycle by blocking innate or adaptive host immune responses or by interfering with the appropriate regulation of apoptosis, cell growth, or DNA replication in infected cells.

In this manuscript, we have extended this earlier work by identifying an additional 14 miRNAs in EBV and by cloning and characterizing 21 miRNAs encoded by the related rhesus lymphocryptovirus (rLCV), a primate virus that is believed to have diverged from EBV ≥13 million years ago [15,16]. We show that both EBV and rLCV encode two clusters of miRNAs, one located near the viral *BHRF1* gene and a second in the *BART* gene. Remarkably, several miRNAs are highly conserved between these two evolutionarily distinct herpesviruses, thus arguing for their importance in the viral life cycle. We also show that the *BHRF1* cluster of EBV miRNAs is selectively expressed in EBV-infected cells undergoing a stage III latent infection, including lymphoblastoid cell lines (LCLs) and passaged Burkitt lymphoma (BL) cells, but is not detected in cells undergoing stage II or stage I EBV latent infection, such as nasopharyngeal carcinoma (NPC) cells. In contrast, the viral *BART* miRNA cluster is highly expressed in NPCs and was also readily detected in an EBV+ primary effusion lymphoma (PEL) cell line, all of which represent EBV stage II latency, but it is barely detectable in LCLs and in most BL cell lines. These data suggest that virally encoded miRNAs may play distinct roles in the EBV-induced transformation of different human target cells.

## Results

### Identification of 14 Novel EBV miRNAs Encoded within the Viral *BART* Gene

Previously, we reported the cloning and analysis of a set of 11 miRNAs encoded by the pathogenic human herpesvirus KSHV that are expressed in the PEL cell line BC-1, which is latently infected by KSHV [12]. BC-1 cells are also latently infected by a wild-type strain of EBV, and we also cloned 222 cDNAs representing EBV miRNAs, out of the 557 cDNA clones of cellular and viral miRNAs obtained in total. These 222 EBV miRNAs consisted of 15 distinct sequences (Table 1) that derived from 13 different predicted primary miRNA stem-loop precursors (Figure S1). Remarkably, only one of these 15 miRNAs was essentially identical in sequence to one of the five EBV-encoded miRNAs previously reported by Pfeffer et al. [11]. Specifically, miR-BART1-5p is identical to the previously reported miR-BART1 miRNA except that it is 2 to 3 nt longer at the 3′ end. This difference may be real or, alternately, the previously reported miR-BART1 cDNA, which was only cloned once, may have suffered a 3′ deletion during cloning. In total, these data indicate that wild-type EBV actually encodes at least 17 different viral miRNAs.

**Table 1:**
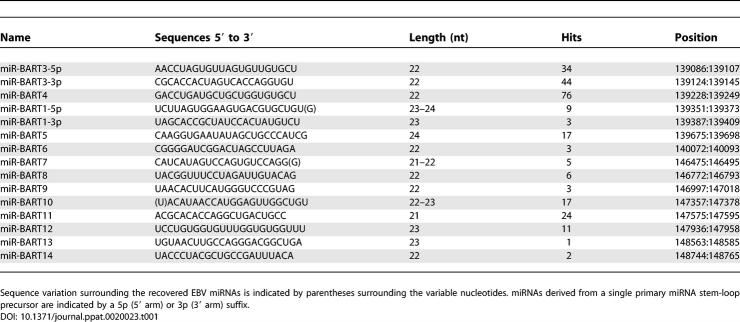
Sequence and Genomic Location of EBV miRNAs Cloned from the BC-1 Cell Line

**Figure 1:**
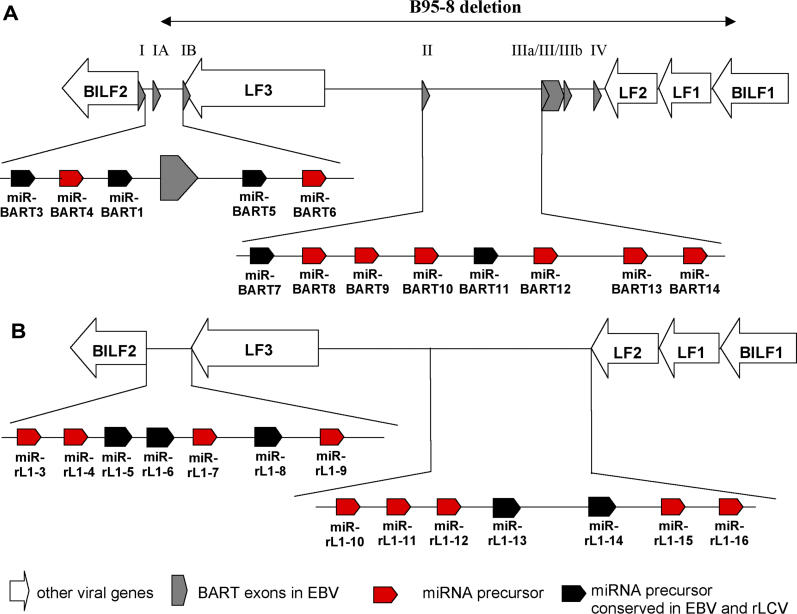
Genomic Location of Selected EBV and rLCV miRNAs (A) Schematic of a segment of the EBV genome, extending from 137,490 to 152,641, with *EBV* genes located on the antisense strand and *BART* mRNA exons located on the sense strand indicated. The location of the EBV *BART* miRNAs identified in this report is indicated. Also shown is the extent of the *BART* gene deletion found in the EBV B95–8 strain. Not shown are the three miRNAs encoded within the EBV *BHRF1* miRNA cluster, which extends from 41,474 to 42,990, or the miR-BART2 miRNA, which is located 3′ to the other *BART* miRNAs between positions 152,747 and 152,768. (B) Schematic of a similar segment of the rLCV genome, extending from position 131,487 to 148,684, with known rLCV homologs of *EBV* genes indicated. The rLCV miRNAs identified in this report are indicated and the miRNAs conserved in EBV highlighted. Although the *BART* gene is thought to be conserved in this region of the rLCV genome, based on sequence analysis, the *BART* exons in rLCV have not been mapped. Not shown are the rLCV miR-rL1-1 and miR-rL1-2 miRNAs, which are encoded 3′ to position 35,323 and 36,709, respectively.

The previous report on EBV miRNAs by Pfeffer et al. [11] isolated and cloned miRNAs from the human BL cell line BL41/95. BL41/95 cells are infected with the EBV B95–8 laboratory isolate that, when compared to wild-type EBV, suffers from an approximately12-kb deletion that removes a large part of the EBV *BART* gene [17,18]. As shown in Figure 1, and implied by the miRNA names listed in Table 1, all of the novel EBV miRNAs identified in this report are derived from a miRNA cluster located within the predicted introns of the *BART* gene, as previously also proposed for miR-BART1 and miR-BART2 [11]. Moreover, miR-BART5 to miR-BART14 are all located within the region deleted in the B95–8 EBV strain, thus explaining their lack of detection by Pfeffer et al. [11]. In contrast, miR-BART3 and miR-BART4 are still present in B95–8 and are in fact located within the same predicted *BART* gene intron as miR-BART1 (Figure 1A), and hence are presumably coexpressed. However, since miR-BART1 was only cloned once [11], it seems possible that these two viral miRNAs were simply missed due to their low expression (see below).

### EBV miRNAs Are Differentially Expressed in Latently EBV-Infected Cells

In contrast to the single miR-BART1 cDNA obtained, Pfeffer et al. [11] cloned several copies of three other EBV miRNAs derived from a second, distinct miRNA cluster adjacent to the *BHRF1* gene. Specifically, these workers cloned miR-BHRF1–1 twice, miR-BHRF1–2 fifty times and miR-BHRF1–3 twenty-three times. Using PCR analysis, we confirmed that the *BHRF1* gene was intact in latently EBV-infected BC-1 cells (unpublished data), and the reason for our inability to clone any of the three previously reported EBV BHRF1 miRNAs was therefore unclear. To address this issue, we analyzed the expression of a selection of EBV miRNAs and mRNAs in the PEL cell line BC-1; in the wild-type EBV-infected BL cell lines Raji, MUTU, Jijoye, and Namalwa; in the EBV strain B95–8-infected BL cell line BL41/95 (used as a source of EBV miRNAs by Pfeffer et al. [11]); in the LCL IM-9; in the NPC cell line C666–1; and finally in the NPC tumor C15, which is passaged as a xenograft in nude mice [19]. BCBL-1, a PEL cell line not infected by EBV, was used as a negative control. This analysis included several BL cell lines that have been extensively passed in culture—i.e., Raji, Jijoye, Namalwa, and MUTU III—all of which represent EBV stage III latency. We also analyzed one BL cell line, MUTU I, that is an early passage variant of MUTU III and has been shown to be in stage I latency [20]. All these EBV-infected cell lines contain type I EBV except for Jijoye, which is infected with a type II EBV [21].

**Figure 2:**
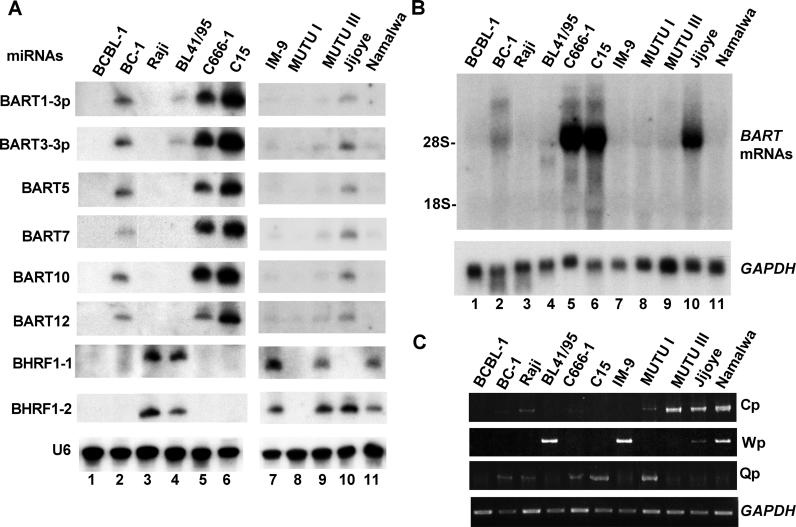
Analysis of EBV miRNA and mRNA Expression in Tumor-Derived Cells (A) Northern analysis of selected EBV miRNAs in total RNA samples derived from the indicated cell lines and tumors. The EBV uninfected PEL cell line BCBL-1 served as a negative control and U6 RNA as a loading control. (B) Northern analysis of *BART* mRNA expression. The total RNA samples analyzed here are the same ones used in (A). The probe used was specific for the invariant exon 7 of the alternatively spliced *BART* miRNAs. *GAPDH* mRNA expression was used as a loading control. The mobility of 28S and 18S rRNA is indicated. (C) RT-PCR analysis using primers specific for transcripts initiating at the viral Cp, Wp, and Qp promoters. This analysis used oligo(dT)-primed cDNA preparations. Primers specific for the cellular *GAPDH* mRNA were used as a control.

As noted above, miRNA analysis using RNA derived from the latently EBV-infected cell line BC-1 resulted in the cloning of the *BART* miRNAs shown in Figure 1A but failed to identify EBV miRNAs derived from the distinct *BHRF1* cluster. Consistent with this cloning result, we observed readily detectable levels of the mature miR-BART1-3p, miR-BART3-3p, miR-BART5, miR-BART7, miR-BART10, and miR-BART12 miRNAs upon Northern analysis of RNA prepared from BC-1 cells, but failed to detect either miR-BHRF1–1 or miR-BHRF1–2 (Figure 2A, lane 2). In contrast, Northern analysis of RNA obtained from the BL41/95 cell line (Figure 2A, lane 4) revealed high-level expression of miR-BHRF1–1 and miR-BHRF1–2 but little or no detectable expression of any of the miRNAs encoded by the EBV *BART* miRNA cluster. While this was predicted for miR-BART5, BART7, BART10, and BART12, all of which are deleted in EBV strain B95–8 (Figure 1A), it was also true for miR-BART1-3p and miR-BART3-3p, both of which are still present in the B95–8 strain and both of which were readily detectable in BC-1 cells.

Analysis of RNA obtained from the BL, LCL, and NPC samples, all of which are infected with wild-type EBV strains, indicated that the LCL and the four BL cell lines largely shared the miRNA expression pattern seen in BL41/95, while the two NPC samples revealed an exaggerated form of the miRNA expression pattern seen in BC-1. Specifically, very high-level expression of all EBV miRNAs derived from the *BART* miRNA cluster was observed in the NPC samples without detectable expression of the miRNAs encoded within the *BHRF1* cluster (Figure 2A, lanes 5 and 6). Conversely, the IM9 LCL and the BL cell lines Raji, MUTU III, and Namalwa had readily detectable expression of miR-BHRF1–1 and miR-BHRF1–2 (Figure 2A, lanes 3, 7, 9, and 11), yet very low to almost undetectable expression of the viral *BART* miRNAs, even though the *BART* gene is intact in all these cell lines. A different miRNA expression pattern was noted in MUTU I, which did not express readily detectable levels of any viral miRNA, and in Jijoye, which expressed high levels of *miR-BHRF1–2,* no detectable *miR-BHRF1–1,* and intermediate levels of all the *BART* miRNAs analyzed (Figure 2A).

To extend this analysis to additional EBV-infected cell lines, we also performed Northern analyses for miR-BART1-3p, miR-BART3-3p, miR-BART7, miR-BART10, miR-BHRF1–1, and BHRF1–2, using RNA derived from the BL cell lines P3HR-1 and Daudi, the LCLs HMy2.CIR and HCC1739 BL, and the PEL cell line JSC-1. P3HR-1 is a subclone of Jijoye containing an EBV that has lost a segment of the viral genome including the *EBNA-2* gene but that should retain all the EBV miRNAs [22]. While HMy2.CIR is a spontaneous LCL, HCC1739 BL is derived by infection of B cells with the EBV B95–8 laboratory strain, which as noted above is deleted for miR-BART5 through miR-BART14 (Figure 1). Analysis of the viral miRNA expression pattern showed that JSC-1 is similar to BC-1, the other PEL cell line tested, in that it expressed the viral BART miRNAs but did not express detectable BHRF1 miRNAs (Figure S2). The LCLs HMy2.CIR and HCC1739 BL were similar to the IM-9 LCL in that they both expressed readily detectable levels of miR-BHRF1–1 and BHRF1–2 but little or no viral BART miRNAs. Daudi was similar to the other BL cell lines examined in Figure 2, in that it expressed readily detectable levels of the viral BHRF1–1 and BHRF1–2 miRNAs but only low levels of the five BART miRNAs analyzed (Figure S2). Finally, P3HR-1, a BL cell line that derives from Jiyoye, shared with Jijoye the property of expressing high levels of miR-BHRF1–2 but no detectable miR-BHRF1–1. However, P3HR-1 differed from Jijoye, and was more similar to the other BL cell lines examined, in that the various viral BART miRNAs were not detectable (Figure S2).

The discordant expression of the miR-BHRF1–1 and miR-BHRF1–2 miRNAs observed in Jijoye and its subclone P3HR-1 was unexpected, given that these miRNAs are located close to each another and show concordant expression in all other EBV-infected cell lines analyzed (Figures 2A and S2). We therefore used PCR to clone and sequence a 418-bp region flanking the *miR-BHRF1–1* sequence from the EBV genome present in Jijoye cells (Figure S3A). This analysis revealed only two single nucleotide sequence differences, neither of which was located in the mature *miR-BHRF1–1* sequence. However, one sequence change maps to the *miR-BHRF1–1* passenger strand and this G to C change is predicted to disrupt a G–C basepair located within the primary miRNA hairpin, thereby generating a 4-nt symmetrical bulge (Figure S3B). Inspection of a wide range of vertebrate miRNA precursors failed to identify any stems containing two symmetrical 4-nt bulges within the miRNA duplex region (unpublished data), and we therefore hypothesize that this 1-nt mutation is disrupting the appropriate processing of the primary *miR-BHRF1–1* transcript in Jijoye and P3HR-1 cells. Whether this mutation is restricted to the EBV isolate present in Jijoye and P3HR-1, or is a general characteristic of EBV type II strains, remains to be established.

### Transcriptional Origin of the EBV miRNAs

The 14 EBV miRNAs encoded within the viral *BART* gene cluster are all located within the predicted introns of this alternatively spliced gene (Figure 1A), and one would therefore predict that the *BART* miRNAs would be coordinately expressed and that the total level of expression of the various alternatively spliced *BART* mRNAs would correlate with the expression level of the *BART* miRNAs. The miRNA expression data presented in Figures 2A and S2 strongly support the hypothesis that the *BART* miRNAs are indeed coordinately expressed. To examine whether *BART* miRNA expression indeed correlates with *BART* mRNA expression, we performed a Northern analysis using the same RNA samples analyzed for miRNA expression in Figure 2A but this time using a probe specific for the invariant exon 7 found in all spliced *BART* mRNAs. As shown in Figure 2B, we indeed observed high-level expression of *BART* mRNA in the NPC cell samples (lanes 6 and 7), an intermediate level of *BART* mRNA expression in Jijoye and BC-1 cells (lanes 2 and 11), and low to almost undetectable expression in the other BL cell lines and in the LCL tested. The *BART* gene has previously been shown to give rise to several alternatively spliced mRNA variants, with a major species at approximately 4.8 kb and a more minor species at approximately 6.2 kb [23–25], and the data presented in Figure 2B are consistent with this prediction. These data also confirm the previous observation [23–27] that *BART* mRNAs are expressed at high levels in NPC cells and at much lower levels in most BL cells, with Jijoye an obvious exception. More important, these data largely confirm the hypothesis that the *BART* mRNA expression pattern (Figure 2B) is predictive of the expression pattern of the entire EBV *BART* miRNA cluster (Figure 2A). We do not currently understand why the BART miRNA expression levels seen in BC-1 and Jijoye are comparable, yet Jijoye expresses a significantly higher level of BART mRNA (Figure 2A and 2B), although we hypothesize that this may reflect less efficient miRNA processing in Jijoye cells.

Previously, Pfeffer et al. [11], who first identified the EBV *BHRF1* miRNA cluster, proposed that these three miRNAs might be coexpressed with mRNAs encoding the *BHRF1* gene product. However, this appears unlikely as *BHRF1* is thought to be first expressed early during lytic replication [28,29], and the hairpin precursor for miR-BHRF1–1 appears to be located 5′ to the cap site for the promoter that drives lytic *BHRF1* mRNA expression. An alternative hypothesis is that the *BHRF1* miRNA cluster is actually processed out of the BamHIH intron present in the very long pre-mRNAs that initiate at the viral Cp and Wp promoters and, when processed, are translated to give rise to the viral EBNA proteins. Transcription from Cp and Wp is characteristic of type III EBV latency [29,30], while EBV-infected cells that are undergoing stage I or II latency instead use the Qp promoter to express EBNA1 [31,32]. Because the Qp promoter differs from the Cp and Wp promoters in being located between the *BHRF1* and *EBNA1* open reading frames, viral pre-mRNAs initiating at Qp could not be processed to yield any of the *BHRF1* miRNAs.

To test whether expression of the *BHRF1* miRNA cluster indeed correlates with the activity of the Cp and/or Wp promoters, we performed RT-PCR using previously described primers [33] specific for RNAs initiating at Wp, Cp, or Qp. As shown in Figure 2C, we saw readily detectable levels of transcription from Cp in Namalwa, Jijoye and MUTU III, with weaker activity in Raji and MUTU I. The Wp promoter was active in Jijoye, Namalwa, IM-9, and BL41/95. Finally the Qp promoter, which is characteristic of stage I or stage II latency, was active in MUTU I, C666–1, C15, BC-1 and, less strongly, in Raji. Therefore, these data show that the cells predicted to be undergoing EBV stage III latency (Raji, BL41/95, IM-9, MUTU III, Jijoye, and Namalwa) all utilize the Cp and/or Wp promoters, while the cells predicted to be undergoing stage I or stage II latency (MUTU I, C666–1, BC-1, and C15) all utilize the Qp promoter to express EBNA1. The observation that Raji is weakly positive for Qp function, while MUTU I is weakly positive for Cp function, likely implies that these BL cell lines are actually a mixture of cells in stage I and stage III latency. Nevertheless, the overall conclusion from these data is that expression of the *BHRF1* miRNA cluster indeed correlates with usage of the Cp and Wp promoters and is therefore likely to be characteristic of stage III latency.

### Expression of Several EBV miRNAs Increases during Lytic Replication

In the case of the miRNAs encoded by the γ herpesvirus KSHV, induction of lytic viral replication fails to significantly enhance the level of expression of ten out of the 11 viral miRNAs, all of which are expressed in latently infected cells [10,12]. The exception to this generalization, miR-K10, appears to be expressed at higher levels during lytic replication because, unlike the other ten viral miRNAs, it lies within a viral transcription unit that is activated by lytic replication [10,12]. Consideration of the genomic location of the viral EBV miRNAs suggests, in contrast, that expression of many of these viral miRNAs is likely to increase after induction of lytic replication. miR-BHRF1–2 and BHRF1–3 lie within the 3′ untranslated region of the early lytic mRNA encoding BHRF1 [11,28,29] and therefore would be expected to be induced during lytic EBV replication. Moreover, recent data demonstrate that BART mRNA expression is also significantly induced during lytic replication of EBV [34], so one would predict that BART miRNA expression would increase in parallel. It is less clear whether lytic replication would be likely to result in increased miR-BHRF1–1 expression, as this viral miRNA appears to lie 5′ to the BHRF1 mRNA transcription start site [11].

**Figure 3:**
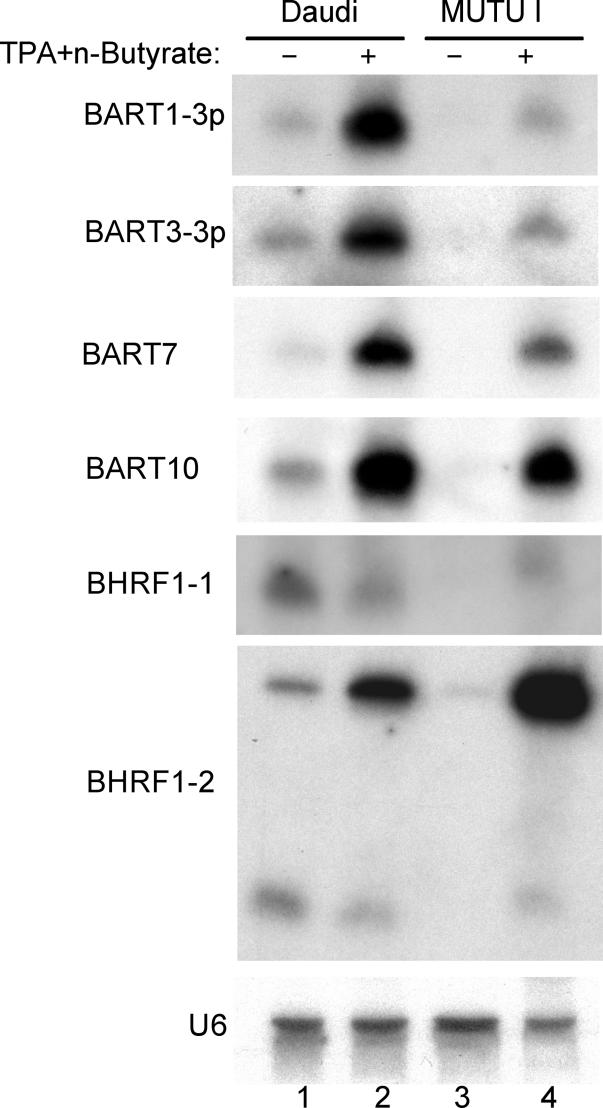
Induction of Lytic Replication Can Increase EBV miRNA Expression The EBV-infected B-cell lines Daudi and MUTU I were either cultured under normal conditions or treated with TPA (30 ng/ml) and n-butyrate (300 ng/ml) for 48 h. At this point cell samples were analyzed for entry into lytic EBV replication by immunofluorescent detection of Zebra expression (see Figure S4) or used for RNA preparation and Northern analysis, as described in Figure 2. In the case of miR-BHRF1–2, we here show a larger panel that also includes the approximately 59-nt pre-miRNA precursor, as this was more readily detected than the mature approximately 23-nt miR-BHRF1–2 miRNA.

Examination of the level of lytic EBV replication induced by treatment of the various LCL, BL, and PEL cell lines analyzed in Figures 2A and S2 with TPA and n-butyrate revealed that Daudi and MUTU I were the most responsive. Specifically, using immunofluorescence to detect the EBV Zebra protein, which is activated very early during lytic replication [29], we observed that TPA/n-butyrate treatment increased the number of Zebra-positive Daudi cells from approximately 1.7% to 19.4%, while the number of Zebra-positive MUTU I cells increased from less than 0.5% to approximately 54% (Figure S3). Analysis of viral miRNA expression revealed that induction of EBV lytic replication resulted in a clear increase in the expression of the viral miRNAs miR-BART1-3p, miR-BART3-3p, miR-BART7, miR-BART10-3p, and miR-BHRF1–2, but did not significantly enhance expression of miR-BHRF1–1 (Figure 3). In the case of miR-BHRF1–2, this increase was particularly apparent when the level of expression of the pre-miRNA was analyzed, although an increase in the mature miR-BHRF1–2 level was also detected in MUTU I cells. Together these data therefore argue that the expression level of several EBV miRNAs increases significantly during lytic replication, with the apparent exception of miR-BHRF1–1.

### Viral miRNAs Have Been Conserved during Lymphocryptovirus Evolution

While between nine and 18 miRNAs are encoded by each of the herpesviruses analyzed so far, none of these viral miRNAs show any obvious sequence homology [10]. On the other hand, the herpesviruses that have been analyzed, i.e., EBV, KSHV, cytomegalovirus, and mouse herpesvirus 68, are either from different herpesvirus genera or, in the case of mouse herpesvirus 68, derived from a very different species.

We reasoned that if virally encoded miRNAs are indeed important for aspects of the virus replication cycle in vivo, then viral miRNAs should tend to be conserved during viral evolution. To address the validity of this hypothesis, we sought to identify miRNAs encoded by rhesus lymphocryptovirus (rLCV), a member of the lymphocryptovirus genus of which EBV is the human representative. The primate LCV genus includes members that infect every primate species examined so far, and the sequence divergence between different primate LCVs predicts a phylogenetic tree that parallels that of the primate species themselves [16]. It has therefore been proposed that primate LCVs have coevolved with their primate host species and that EBV and rLCV evolutionarily diverged up to 23 million years, and at least 13 million years ago [16]. Sequence analysis of rLCV shows that this primate virus has approximately 65% overall nucleotide homology with EBV, with structural proteins being highly conserved, while genes expressed during EBV latent infection are much less well conserved [15]. More important, this analysis predicts that the EBV *BHRF1* and *BART* genes are both conserved in rLCV.

**Figure 4:**
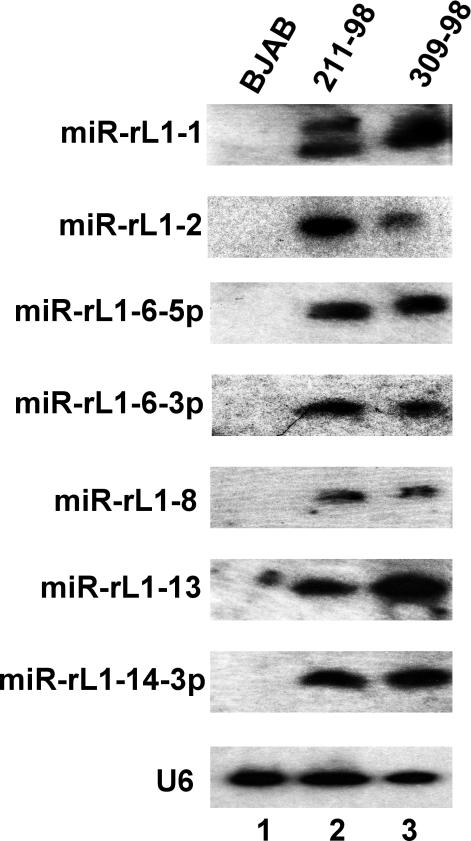
Analysis of rLCV miRNA Expression Northern analysis of selected rLCV miRNAs in the rLCV-infected rhesus B-cell lines 211–98 and 309–98. The human B-cell line BJAB served as a negative control.

We performed cDNA cloning of rLCV miRNAs using RNA derived from the latently infected rhesus B cell line 211–98 [35]. As shown in Table 2, 257 rLCV miRNA clones representing 21 distinct viral miRNA sequences were obtained. These could be further assigned to 15 different primary miRNA stem-loop precursors (Figure S5). One of these miRNAs, miR-rL1-1, was derived from a region adjacent to the rLCV *BHRF1* gene, while the other 20 rLCV miRNAs were derived from the rLCV *BART* locus. Indeed, as shown in Figure 1B, the latter rLCV miRNAs are located at the same genomic position, relative to the viral *LF2, LF3,* and *BILF2* genes encoded on the opposite DNA strand, as the EBV *BART* miRNA cluster. As shown in Figure 4, expression of rLCV miRNAs could be readily demonstrated in the latently rLCV infected 211–98 cell line as well as in a second, unrelated latently rLCV infected cell line, termed 309–98 [35]. All the rLCV miRNAs analyzed gave rise to a single band in this Northern analysis except miR-rL1-1.

**Table 2:**
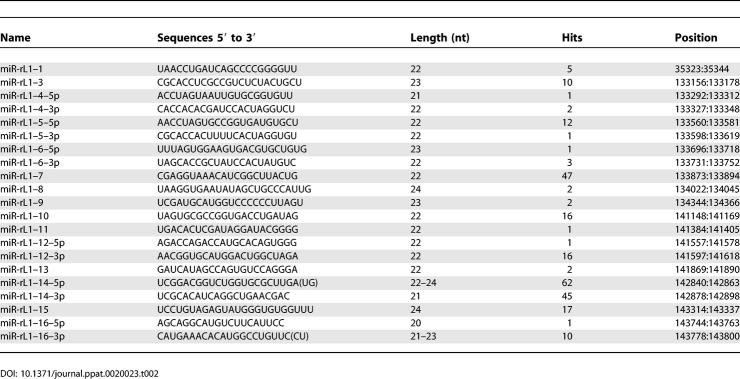
Sequence and Genomic Location of rLCV miRNAs Cloned from the 211–98 Cell Line

**Figure 5:**
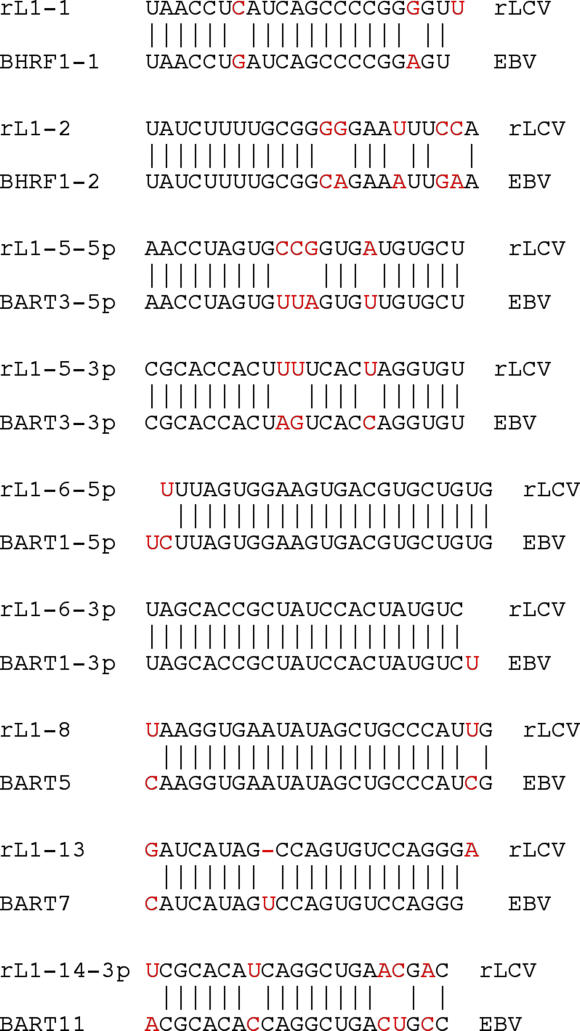
Sequence Comparison of miRNAs That Are Evolutionarily Conserved in EBV and rLCV and Expressed in Virus-Infected Cells All the indicated miRNAs were cDNA cloned from infected cells except miR-rL1-2, whose existence was predicted in silico and then confirmed by Northern analysis (Figure 4).

Sequence comparison of the EBV and rLCV miRNAs revealed that eight miRNAs, derived from six different stem-loop precursors, have been largely or entirely conserved during the evolutionary divergence of rLCV and EBV, especially in the miRNA “seed” region (position 2 to 8 from the 5′ end; Figure 5). Moreover, the genomic 5′ to 3′ order of the EBV *BART* miRNAs that are conserved in rLCV is unchanged in this distantly related primate lymphocryptovirus (Figure 1). The small number of miRNA sequence changes that are observed are generally either at the very ends of these miRNAs, which are known to contribute only minimally to target mRNA recognition [36], or represent C to U or G to A changes that may imply basepairing to a G or to a U residue, respectively (Figure 5). Other sequence changes that do imply a real difference in basepairing, e.g., a G to C difference between the EBV miR-BHRF1–1 and the rLCV miR-rL1-1 miRNA, may reflect a real difference in the mRNA target sequence in humans versus rhesus macaques. Of particular interest is the one nucleotide insertion seen upon comparison of EBV miR-BART7 with rLCV miR-rL1-12, while all other viral miRNA pairs show neither insertions nor deletions (Figure 5). This may imply that the miR-rL1-12/miR-BART7 miRNA pair targets a noncoding region, such as an mRNA 3′ untranslated region, that can readily tolerate a 1-nt deletion or insertion.

**Figure 6:**
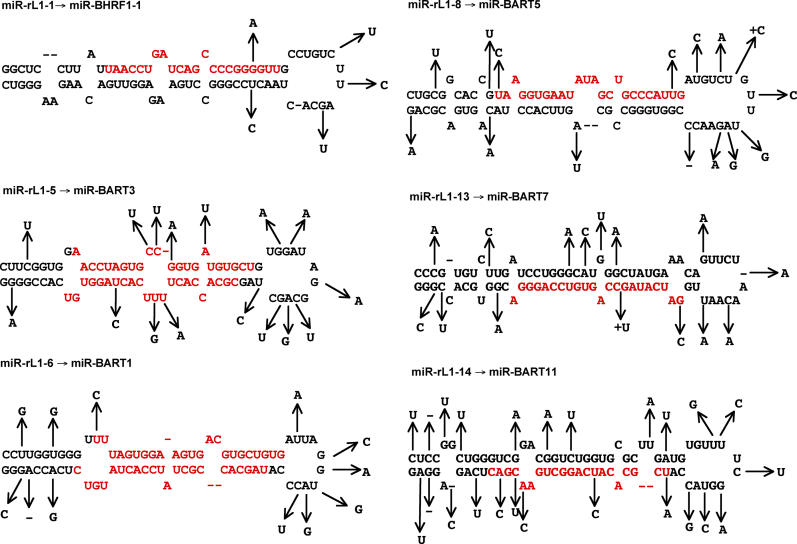
Sequence Comparison of the Predicted Primary miRNA Stem-Loop Structures of the Indicated rLCV and EBV miRNAs The stem-loop shown is the rLCV sequence, with the changes observed in EBV indicated. The mature miRNA sequences are shown in red. In some cases, the miRNA precursors give rise to two mature miRNAs. A “+” sign indicates an insertion.

It could be argued that the sequence conservation of the EBV and rLCV miRNAs documented in Figure 5 reflects the conservation of longer stretches of viral DNA sequence. Conversely, if this conservation is functionally important, then closely adjacent sequences might show more extensive sequence divergence. To examine this issue, the sequences of the predicted primary miRNA stem-loop precursors for each of these EBV and rLCV miRNAs were compared. Previously, we and others have reported that these RNA stem-loops consist of at least three distinct domains [36,37]. The central domain consists of the approximately 22-nt mature miRNA sequence, shown in red in Figure 6, and its complement, termed the miRNA passenger strand, that forms part of the approximately 22-bp miRNA duplex intermediate, but is generally not incorporated into the RNA-induced silencing complex, or is incorporated less efficiently. The terminal loop is a large (≥10-nt) unstructured loop (although RNA folding programs may predict a smaller loop adjacent to a short, rather unstable stem) whose sequence appears irrelevant as long as it maintains an open structure [37]. Finally, the base of the stem consists of an approximately 8- to 10-bp helical extension of the miRNA duplex that is critical for efficient nuclear processing of the primary miRNA precursor. Because this sequence does not form part of the miRNA duplex intermediate per se, its sequence is not important in and of itself, although maintenance of a helical structure is required [4,38]. The approximately 80-nt primary miRNA stem-loop structure is in turn flanked by largely nonstructured RNA sequences that are not believed to play a sequence-specific role in miRNA processing and expression [39]. Therefore, we would predict that, even though the mature viral miRNA sequences are well conserved (Figure 5), the flanking basal stem and, particularly, the terminal loop and adjacent single-stranded RNA regions should show significantly more sequence variation.

In Figure 6, the RNA sequences of six predicted primary stem-loop precursors of miRNAs that are conserved in EBV and rLCV are compared. As may be observed, there is indeed better conservation of the mature miRNA sequence when compared to the flanking basal stem and, particularly, the terminal loop. Overall, the sequences of the six miRNA duplex intermediates are approximately 89% conserved between EBV and rLCV, the basal stems are approximately 84% conserved, the terminal loops are approximately 62% conserved, while the adjacent unstructured flanking sequences, extending approximately 90 nt each side of the predicted miRNA stem-loops shown in Figure 6, are approximately 68% conserved. This compares to an overall sequence conservation of 65% between the EBV and rLCV genomes [15]. The statistical significance of the observed conservation in EBV and rLCV of the pre-miRNA stem extension, the miRNA duplex, and the pre-miRNA terminal loop sequence was analyzed using the paired t-test with the null hypothesis being that these sequences are not more highly conserved than the 5′ and 3′ 100-nt segments flanking each viral pre-miRNA. In fact, both the miRNA duplex regions and the miRNA extended stem regions were found to be significantly more highly conserved from EBV to rLCV than either the terminal loop or the flanking sequences (*p* < 0.05).

Additional computer analysis of the rLCV genome sequence revealed that this virus contains a sequence that is identical at 18 out of 23 positions to the mature EBV miR-BHRF1–2 miRNA (Figure 5). Moreover, this sequence is found at the same relative genomic position in rLCV, i.e., immediately 3′ to the BHRF1 open reading frame. This candidate rLCV miRNA, termed miR-rL1-2, also forms part of a predicted RNA hairpin that is closely similar to the RNA hairpin predicted for the primary EBV miR-BHRF1–2 precursor (Figure S6). To test whether this candidate rLCV miRNA is expressed in latently infected cells, we performed a Northern analysis that confirmed the expression of miR-rL1-2 in rLCV-infected 211–98 and 309–98 cells but not in control, uninfected cells (Figure 4). It therefore appears that rLCV encodes an additional miRNA, missed during cDNA cloning, that is closely similar to EBV miR-BHRF1–2. This brings the number of distinct miRNAs conserved during the evolutionary divergence of EBV and rLCV to at least seven.

## Discussion

### Lymphocryptovirus miRNA Conservation and Function

Recent reports have documented the existence of miRNAs encoded within the genomes of several herpesviruses, including five miRNAs in EBV, all of which are expressed in latently infected cells [10–13]. This report extends these earlier data by (1) documenting that wild-type EBV actually encodes at least 17 miRNAs, ( 2) showing that these viral miRNAs are derived from two distinct miRNA clusters that are differentially expressed in latently EBV-infected cells, and (3) showing that several EBV miRNAs have been conserved across ≥13 million years of primate lymphocryptovirus evolution.

Analysis of EBV miRNA expression has demonstrated the existence of a three-miRNA cluster located adjacent to the viral *BHRF1* gene [11] and a second cluster of 14 miRNAs located within the viral *BART* gene (Figure 1A; Table 1). Similarly, analysis of viral miRNA expression in rLCV latently infected cells identified at least 16 distinct miRNAs, two encoded adjacent to the rLCV *BHRF1* homolog and the other 14 in the rLCV *BART* gene locus (Figure 1B; Table 2). Of these, nine miRNAs, derived from seven different precursor stem-loops, have been largely conserved across the >13 million years of evolution that separate these two primate lymphocryptoviruses (Figure 5) [16]. This conservation is clearly statistically significant, and a comparison of the similarity of closely adjacent viral sequences—e.g., those that form the terminal loop of the viral primary miRNA precursors—reveals far less sequence conservation than seen in the viral miRNAs themselves (Figure 6). So, what does this evolutionary conservation tell us about EBV miRNA function?

One possibility raised by this high level of sequence conservation is that some of these viral miRNAs are actually acting as small interfering RNAs—i.e., inducing target mRNA degradation—rather than as canonical vertebrate miRNAs—i.e., blocking target mRNA translation. Translational inhibition requires only fairly modest miRNA sequence complementarity to the mRNA target, most notably in the approximately 8-nt “seed” region located near the 5′ end of the miRNA, while mRNA cleavage requires extensive homology to the target mRNA [36,40–44]. The almost complete conservation of several of these viral miRNAs (Figure 5) therefore could argue for a degradative mechanism. On the other hand, it is also possible that each of these conserved viral miRNAs is partially complementary to multiple cellular mRNAs and that the observed sequence conservation is mandated by a requirement to maintain significant complementarity to several targets that are subject to translational inhibition.

The extensive conservation of these viral miRNAs suggests that their mRNA targets are likely to be predominantly cellular rather than viral. EBV and rLCV show significant sequence divergence, especially in the viral latent genes [15], and one would predict that a putative viral mRNA target sequence and its viral miRNA complement would coevolve over time. This is indeed what is seen in the flanking basal stems of the viral miRNA precursors, which show more sequence divergence between EBV and rLCV than do the miRNAs themselves, yet retain their ability to form an RNA duplex (Figure 6). In contrast, if the target mRNA is cellular, and hence fairly invariant, then very little viral miRNA sequence change could be tolerated. Moreover, with the exception of miR-BART2, which is antisense to the EBV BALF5 mRNA and has been proposed to regulate BALF5 expression by a degradative RNA interference mechanism [11], none of the EBV miRNAs are, in fact, located opposite known viral exons (Figure 1A). Rather, they are found opposite introns or noncoding sequences.

While seven of the lymphocryptovirus miRNAs are largely conserved in terms of both sequence and relative genomic position in EBV and rLCV, the remaining approximately nine miRNAs are not. We believe this is not surprising given the short approximately 22-nt size of mature miRNAs, and given that some sequence differences with the mRNA target can be tolerated [36]. As a result, one might expect potentially very rapid evolution of viral miRNAs, resulting in the selection of novel cellular mRNA targets whose down-regulation is advantageous to the virus. More surprising, in our view, is the fact that several of the lymphocryptovirus miRNAs have been conserved across millions of virus replication cycles.

### Differential Expression of EBV miRNAs

In addition to the functional implications suggested by the observed evolutionary conservation of these lymphocryptovirus miRNAs, our analyses also provide insights into the regulation of EBV miRNA expression. The initial report [11] identifying the miR-BHRF1–1, BHRF1–2, and BHRF1–3 miRNAs, as well as miR-BART1 and miR-BART2, suggested that these two *BART* miRNAs were derived from introns in the *BART* mRNA, as indeed confirmed in this report, while the three miRNAs that form the *BHRF1* miRNA cluster were proposed to be derived from the mRNA encoding the *BHRF1* open reading frame, which is actually thought to be expressed early during replicative infection [28,29]. However, our data show that the *BHRF1* miRNA cluster is only detectably expressed in BL and LCL samples (Raji, BL41/95, IM-9, MUTU III, Jijoye, and Namalwa) that use the Wp and Cp promoters characteristic of type III latency to express the *EBNA* genes [29,30] (Figure 2). In contrast, no miRNAs derived from the *BHRF1* miRNA cluster were detected in samples that were derived from cells in type II EBV latency (the NPCs C666–1 and C15 and the PEL cell lines BC-1 and JSC-1) or type I latency (MUTU I) that use the Qp promoter to express EBNA1 (Figure 2) [31,32]. This correlation of *BHRF1* miRNA cluster expression and Cp/Wp promoter usage suggests that the *BHRF1* miRNAs are actually processed out of the BamHI H intron present in the very long EBNA pre-mRNAs that are transcribed from the Cp and Wp promoters and not, at least during latent infection, from mRNAs encoding *BHRF1.* We therefore propose that expression of the EBV *BHRF1* miRNA cluster is likely to be a characteristic of type III EBV latency. Nevertheless, the observation that miR-BHRF1–2 expression is markedly enhanced after induction of lytic replication (Figure 3) suggests that the BHRF1 mRNAs induced early during productive infection can also function as a pri-miRNA precursor [11]. The observation that the expression of most EBV miRNAs is enhanced during lytic replication distinguishes EBV from KSHV, where the majority of the viral miRNAs show at most a modest increase in expression after induction of lytic replication [10,12].

In this report, we have documented the existence of at least 12 novel EBV miRNAs all of which, like the previously reported miR-BART1 and miR-BART2, are located in the predicted introns of the alternatively spliced mRNAs derived from the EBV *BART* gene (Figure 1A; Table 1). We present data arguing that the various *BART* miRNAs are therefore coordinately expressed and that their abundance is largely predicted by the level of expression of the *BART* mRNAs (Figure 2). The *BART* mRNAs were first identified and shown to be readily detectable in NPC samples but not in EBV-infected lymphoid cell lines, although subsequent studies identified the spliced *BART* mRNAs by RT-PCR in most samples analyzed [18,23,26,27,45,46]. However, the data presented here reveal that the *BART* miRNAs are only readily detected in samples that express significant levels of *BART* mRNAs as determined by Northern blotting, including the C666 and C15 NPC samples and B cell lines BC-1 and Jijoye. We note, however, that low levels of the *BART* miRNAs can be detected in essentially all the cell lines analyzed upon prolonged exposure (unpublished data and [11]). Therefore, while the NPC samples clearly express higher levels of both the *BART* mRNAs and miRNAs than any of the EBV-infected B cells, EBV-infected B cells do appear to express low but detectable levels of both. Overall, these data therefore indicate that expression of the *BART* miRNA cluster is also characteristic of latent EBV infection and suggest that the *BART* miRNAs, like the *BART* mRNAs, may be preferentially expressed in EBV-infected epithelial cells and hence may play a particularly important role during EBV infection of this differentiated cell type. In this context, it is interesting to note that while the EBV *BART* gene is clearly dispensable for transformation of B lymphocytes in vitro [47], a region of the EBV genome that includes the *BART* gene, but excludes known EBV transforming genes such as *LMP1,* has been reported to immortalize primate epithelial cells in culture [48]. It remains to be established whether the miRNAs encoded within the *BART* miRNAs cluster play a role in epithelial cell transformation by EBV.

## Materials and Methods

### Cell culture, tumors, and RNA preparation.

The various PEL, BL, and LCL cell lines analyzed in this report [12,20,21,49,50] were maintained in RPMI 1640 supplemented with 10% or 20% fetal bovine serum, glutamine, and antibiotics. Where necessary, TPA (Sigma, St. Louis, Missouri, United States; final concentration 30 ng/ml) and n-butyrate (300 ng/ml) were added for 48 h prior to RNA preparation. The C15 nasopharyngeal tumor was passaged in nude mice as a xenograft as described previously [19]. The rLCV latently infected rhesus macaque cell lines 211–98 and 309–98 [35] were maintained in RPMI containing 20% fetal bovine serum, 2 mM glutamine, antibiotics, and 10 mM Hepes. The 211–98 cells latently coinfected with rhesus rhadinovirus (RRV) were maintained similarly and were used as a source of RNA for miRNA cloning. All total RNA samples were prepared using TRIzol reagent (Invitrogen, Carlsblad, California, United States). No difference in the pattern of rLCV miRNA expression was detected in 211–98 cells coinfected with RRV, and no RRV-specific miRNAs were cloned from the coinfected cells (unpublished data). The cloning and sequencing of cDNA copies of small RNA species derived from BC-1 cells dually infected with KSHV and EBV, or from 211–98 cells dually infected with rLCV and RRV, was performed as previously described [12,51] using 750 μg of total RNA as starting material. Sequence coordinates for viral miRNAs are given relative to the full-length type I EBV genome sequence or rLCV sequence (see Accession Numbers section).

### Northern blots and RT-PCR analyses.

Northern blots and RT-PCR analyses were conducted as previously described [52]. Briefly, 30 μg of total RNA per sample was used in the miRNA Northern analyses. The specific probe for each miRNA was a 32p-end-labeled full-length antisense DNA oligonucleotide. The probe used for the U6 RNA Northern analysis has been described [12]. For the Northern analysis of *BART* mRNA expression, 10 μg of total RNA per sample was used. A synthetic DNA oligonucleotide antisense to part of *BART* exon 7.

(5′-AGACCCGCGCCTCTACATCACCTCTGTGCCCTGCTGGCGCTGTGTGGGCGA GCTGATGGTTCTGCCCAACCACGGCAA-3′) was used as the specific probe for *BART* mRNA expression. The same blot was stripped and hybridized with a GAPDH mRNA-specific cDNA probe [52].

cDNAs were prepared using RNA samples isolated from each cell line, as previously described [52], using oligo(dT) primers. RT-PCR analyses were performed as described previously [52], using 25 cycles for the GAPDH primers and 40 cycles for the EBV primers. The primers used for detection of GAPDH mRNA [52] and for transcripts initiating at the Cp, Wp, or Qp EBV promoters have been described [33].

## Supporting Information

Figure S1 Predicted Primary miRNA Stem-Loop Structures for the Indicated EBV miRNAs Cloned in This Report The mature miRNAs are indicated in red. RNA folding was performed using MFOLD.
(5.9 MB TIF)

Figure S2 Northern Analysis of EBV miRNA Expression This analysis was performed as described in Figure 2, using total RNA samples derived from the indicated EBV-infected cell lines. U6 was used as a loading control. In the case of miR-BHRF1–2, we present a larger panel that shows both the mature 23-nt miRNA and the approximately 59-nt pre-miRNA.
(1.5 MB TIF)

Figure S3 A Single Nucleotide Mutation May Disrupt *miR-BHRF1–1* Processing in Jijoye Cells (A) Sequence comparison of the genomic region flanking the mature *miR-BHRF1–1* sequence in the type II EBV present in Jijoye cells with the wild-type type I EBV sequence [18]. Two single nucleotide changes and the mature *miR-BHRF1–1* sequences are highlighted in blue.
(B) One of these single nucleotide changes is predicted to disrupt the base-pairing of the stem of the primary *miR-BHRF1–1* precursor, as indicated.
(5.8 MB TIF)

Figure S4 Immunofluorescent Detection of the EBV Zebra Protein Daudi and MUTU I cells were either cultured as normal or induced with TPA (30 ng/ml final concentration) and n-butyrate (300 ng/ml final concentration) for 48 h. At this stage, the cells were either used for RNA analysis (see Figure 3) or fixed and stained using a mouse monoclonal anti-Zebra antibody (Argene Inc., North Massapequa, New York, United States) followed by a TRITC-conjugated donkey antimouse secondary antibody. Cells were also stained with DAPI. In each case, we show a phase image (upper left), DAPI fluorescence (upper right), TRITC fluorescence (lower left), and superimposed DAPI and TRITC fluorescence (lower right). Quantitation of the number of TRITC-positive cells showed that uninduced Daudi were approximately 1.7% Zebra-positive, induced Daudi approximately 19.4% Zebra-positive, uninduced MUTU I less than 0.5% Zebra-positive, and induced MUTU I approximately 54% Zebra-positive.
(5.7 MB TIF)

Figure S5 Predicted Primary miRNA Stem-Loop Structures for the Indicated rLCV miRNAs Mature viral miRNAs are indicated in red.
(6.3 MB TIF)

Figure S6 Sequence Comparison of miR-BHRF1–2 miRNA and miR-rL1-2 Differences between the predicted miR-BHRF1–2 pri-miRNA stem-loop structure shown, and the predicted miR-rL1–2 sequence, are indicated. This rLCV miRNA is encoded 3′ to the rLCV BHRF1 open reading frame, i.e., in the same genomic location as miR-BHRF1–2. Although it was not recovered during cDNA cloning (Table 2), it is detectable in rLCV-infected cells by Northern blot (Figure 4).
(493 KB TIF)

### Accession Numbers

Accession numbers for the EBV genome sequence (AJ507799) and for the rLCV sequence (AY037858) are found at GenBank (http://www.ncbi.nlm.nih.gov/Genbank). The sequences of the novel EBV and rLCV miRNAs and pre-miRNAs described in this report have been deposited in miRBase (http://microrna.sanger.ac.uk/sequences/index.shtml). The EBV miRNAs miR-BART3 to miR-BART14 have been assigned accession numbers MI0003725 through MI0003736. The rLCV miRNAs miR-rL1-1 to miR-rL1-16 have been assigned accession numbers MI0003737 to MI0003752.

## Abbreviations

BL: Burkitt lymphoma
EBV: Epstein–Barr virus
KSHV: Kaposi sarcoma-associated herpesvirus
LCL: lymphoblastoid cell line
miRNA: microRNA
NPC: nasopharyngeal carcinoma
PEL: primary effusion lymphoma
rLCV: rhesus lymphocryptovirus
